# Ester Formation in Alcohol Microdroplet Sprays: Enhanced Reactivity of C_8_ to C_16_ Carboxylic Acids with C_1_ to C_3_ Alcohols and the Effect of Water

**DOI:** 10.1021/acs.jpca.5c04566

**Published:** 2025-11-17

**Authors:** Lincoln Mehndiratta, Justin Wang, Jonathan H. Slade, Vicki H. Grassian

**Affiliations:** Department of Chemistry & Biochemistry, 8784 University of California San Diego, 9500 Gilman Dr, San Diego, California 92037, United States

## Abstract

Esterification reactions are key reactions in organic synthesis with significant industrial and environmental implications. Esterification reactions typically require acids or solid catalysts and/or heat to overcome the activation energy barrier for this condensation reaction. Here, we demonstrate a new approach facilitated by organic acid–alcohol microdroplets formed from bulk solutions of carboxylic acids (C_8_, C_9_, C_12_, and C_16_) dissolved in an excess of methanol, ethanol, or isopropanol. Utilizing a Collison nebulizer, a spray of microdroplets is produced and then deposited onto a CaF_2_ substrate and analyzed with Optical-Photothermal Infrared (O-PTIR) spectroscopy. Following evaporation of the alcohol solvent and water byproduct, individual 1 to 15 μm-sized single particle residues were analyzed. Our analysis reveals ester formation in the microdroplet, as indicated by an intense band at 1745 cm^–1^ in the O-PTIR spectrum of individual particles. This band is due to the characteristic C=O stretching vibrational mode of the ester. Confirmation of the ester product is also seen with high-resolution mass spectrometry. Although no ester formation occurs in the bulk solution, the reactivity within the microdroplet spray suggests that the reaction occurs at the microdroplet–air interface. Furthermore, our results indicate that under ambient conditions, the shorter-chain acids (C_8_, C_9_) undergo complete esterification with near-quantitative yields. In contrast, longer-chain acids (C_12_, C_16_) undergo partial esterification with some remaining carboxylic acid present. The addition of water to the alcohol solution significantly suppresses ester formation in the microdroplet sprays due to the deprotonation of the organic acid and a decrease in organic acid solubility. Overall, this study highlights a potentially efficient method for ester synthesis for shorter-chain esters, eliminating the need for external catalysts or thermal activation. These findings open new possibilities for chemistry applications by leveraging enhanced reaction kinetics observed for reactions in microdroplet alcohol sprays.

## Graphical abstract

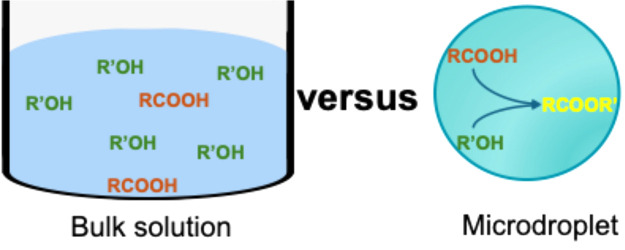

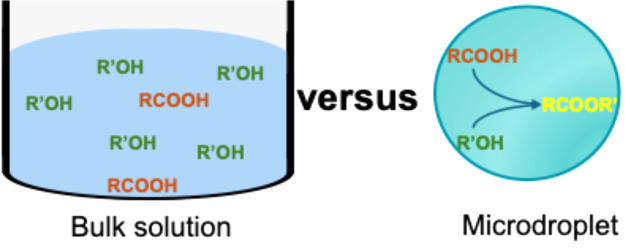

## Introduction

Numerous studies have reported that chemical reactions confined within aqueous micro- and nanodroplets occur at rates far exceeding those observed in macroscopic bulk solutions.^1–9^ This accelerated microdroplet chemistry has important implications across diverse fields, including atmospheric chemistry, organic synthesis, and the origins of life.^10–13^ The enhanced reactivity in aqueous microdroplets has been attributed to the distinct properties of the air–water interface.^14–16^ Due to their surface-to-volume ratios, which are orders of magnitude greater than those of bulk solutions, microdroplets exhibit interfacial kinetics and thermodynamics that can play a dominant role in governing chemical reactions.^17,18^ In addition to their high surface-to-volume ratio, microdroplets are inherently more dynamic than bulk systems and can exhibit rapid gas-droplet partitioning during reactions.^19,20^ Multiple factors have been proposed to influence the acceleration of reactions in microdroplets, including rapid solvent evaporation, partial solvation at the air–liquid interface, physical confinement within small volumes, and the presence of surface charges generated during electrospray ionization (ESI) or electrosonic spray ionization (ESSI) mass spectrometry.^8,21,22^ However, a recent review emphasizes that microdroplets are inherently complex, multiphase systems and that a more comprehensive suite of complementary measurements is required to fully resolve the mechanisms governing microdroplet reactivity.^23^

Esterification is a fundamentally important synthetic chemical reaction step in which a carboxylic acid reacts with an alcohol to produce an ester and water as a byproduct (as shown in eq 1). This reaction is widely utilized in various industries due to the broad applicability of organic esters. Esters play a crucial role in the food and paint industries, as well as in pharmaceuticals and as plasticizers.^24–28^ They serve as preservatives in food products, in the formulation of personal care products, and scent components in soaps and cosmetics.^29–31^ Esters have also gained increased attention due to their application as biodiesel, attributed to their favorable physicochemical properties and renewable nature.^32,33^


1

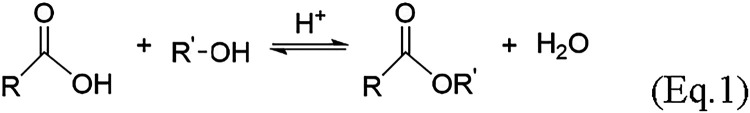




Esterification can occur in the absence of a catalyst, relying on the inherent weak acidity of carboxylic acids. However, the reaction proceeds at a significantly slow rate (reaction conversion was four times higher in the presence of the catalyst compared to the absence of the catalyst), often requiring heating.^34^ The addition of a catalyst, such as dissolved acid (e.g., H_2_SO_4_ or HCl) or solid acid (e.g., sulfonic resins), significantly enhances the reaction rate by facilitating protonation and nucleophilic substitution.^33^ The esterification reaction reaches a dynamic equilibrium after a certain period, beyond which the reaction no longer proceeds significantly unless the equilibrium is shifted. This can be achieved by either adding an excess of alcohol or by continuously removing the water product, thereby driving the reaction forward and promoting greater ester formation.

Previous studies have explored different methods to achieve esterification without the use of a catalyst.^35–41^ One such approach involves noncatalytic thermal esterification, which leverages supercritical alcohols as reaction media. In the supercritical state, alcohols exhibit properties of both liquids and gases, enabling enhanced solubility, diffusion, and mass transfer. This unique phase behavior facilitates esterification by decreasing the activation energy and promoting efficient molecular interactions without the need for an external catalyst.^35,36^ Ester synthesis has also been achieved using either enzymatic catalysis at lower temperatures^42^ or high-temperature thermal processes.^37–39^

Efforts to enhance the efficiency of esterification reactions are focused on addressing inherent limitations. For example, esterification exhibits a low reaction rate due to equilibrium constraints, slow reaction kinetics, and the accumulation of water as a byproduct, which inhibits further ester formation. Additionally, the poor miscibility of reactants can lead to the formation of a thin interfacial film, which restricts mass transfer and further reduces reaction efficiency.^43^ The reaction efficiency can be enhanced by reducing interfacial film thickness through shear mixing, increasing temperature and pressure, or applying ultrasonication to improve mass transfer.^44^ However, these approaches significantly increase energy consumption and operational costs, making the process less economically viable.^45,46^

More recently, microbubble-mediated esterification has been explored. This method utilizes microbubbles generated within a bubble reactor to enhance reaction efficiency.^47,48^ This approach offers several advantages, including lower surface energy, an increased surface-to-volume ratio, elevated internal pressure, and enhanced surface tension. These factors collectively contribute to a larger interfacial area and improved mass transfer and accelerated the reaction rate. Similarly, alcohol microdroplets containing carboxylic acids can achieve some similar advantages and here, we show that microdroplets produced utilizing a Collison nebulizer provide a novel synthesis method for ester formation that operates without the need for a catalyst or heating. Esterification reactions of nebulized microdroplets of medium and long-chain fatty acids including C_8_, C_9_, C_12_, and C_16_ acids dissolved in methanol, ethanol or isopropyl alcohol (IPA) were studied. We also investigate the influence and inhibition that water has on the esterification reaction.

## Materials and Methods

### Materials and Chemicals

All chemicals were commercially purchased and utilized for analysis as received without further purification including carboxylic acids of varying chain lengths, methyl esters, and sodium carboxylates. These included octanoic acid (C_8_H_16_O_2_, Sigma-Aldrich, ≥98%), nonanoic acid (C_9_H_18_O_2_, Sigma-Aldrich, ≥97%), lauric acid (C_12_H_24_O_2_, Acros Organics, 99%), palmitic acid (C_16_H_32_O_2_, Sigma-Aldrich, ≥99%), methyl octanoate (C_9_H_18_O_2_, Sigma-Aldrich, ≥99.8%), methyl nonanoate (C_10_H_20_O_2_, Sigma-Aldrich, ≥99.8%), methyl laurate (C_13_H_26_O_2_, Sigma-Aldrich, 99.5%), methyl palmitate (C_17_H_34_O_2_, TCI Chemicals, ≥99.5%), sodium octanoate (C_8_H_15_NaO_2_, TCI Chemicals, ≥99%), sodium nonanoate (C_9_H_17_NaO_2_, TCI Chemicals, ≥98%), sodium laurate (C_12_H_23_NaO_2_, TCI Chemicals, ≥97%), and sodium palmitate (C_16_H_31_NaO_2_, TCI Chemicals, ≥97%). Additional chemicals used in the study include different alcohols; methanol (CH_3_OH, Fischer chemical, HPLC grade), ethanol (C_2_H_5_OH, Koptec, 200 proof), isopropyl alcohol (IPA) (C_3_H_7_OH, Fischer chemical, HPLC grade), and Milli-Q ultrapure water (Millipore Sigma, ≥18.2 MΩ).

### Optical-Photothermal Infrared (O-PTIR) Spectroscopy

A mIRage instrument (Photothermal Spectroscopy Corp.; Santa Barbara; CA) was used for O-PTIR spectroscopy. The mIRage system enables the simultaneous acquisition of O-PTIR and Raman spectra of single substrate-deposited microdroplets as shown in the simplified schematic presented in Figure 1. This system has been previously employed for the analysis of laboratory-generated and ambient aerosols^49–51^ and has been described in detail in Olson et al.^49^ Briefly, the O-PTIR system utilizes a Cassegrain reflective objective (10× and 50×) to precisely coalign the tunable IR laser and the continuous-wave visible laser (532 nm) onto a particle. When the IR laser interacts with a particle at its resonant IR frequency, the absorbed IR radiation induces a localized photothermal expansion. This leads to a thermal lensing effect that alters the local refractive index and subsequently affects the propagation of the probe beam intensity thereby generating the O-PTIR signal.

**Figure 1:**
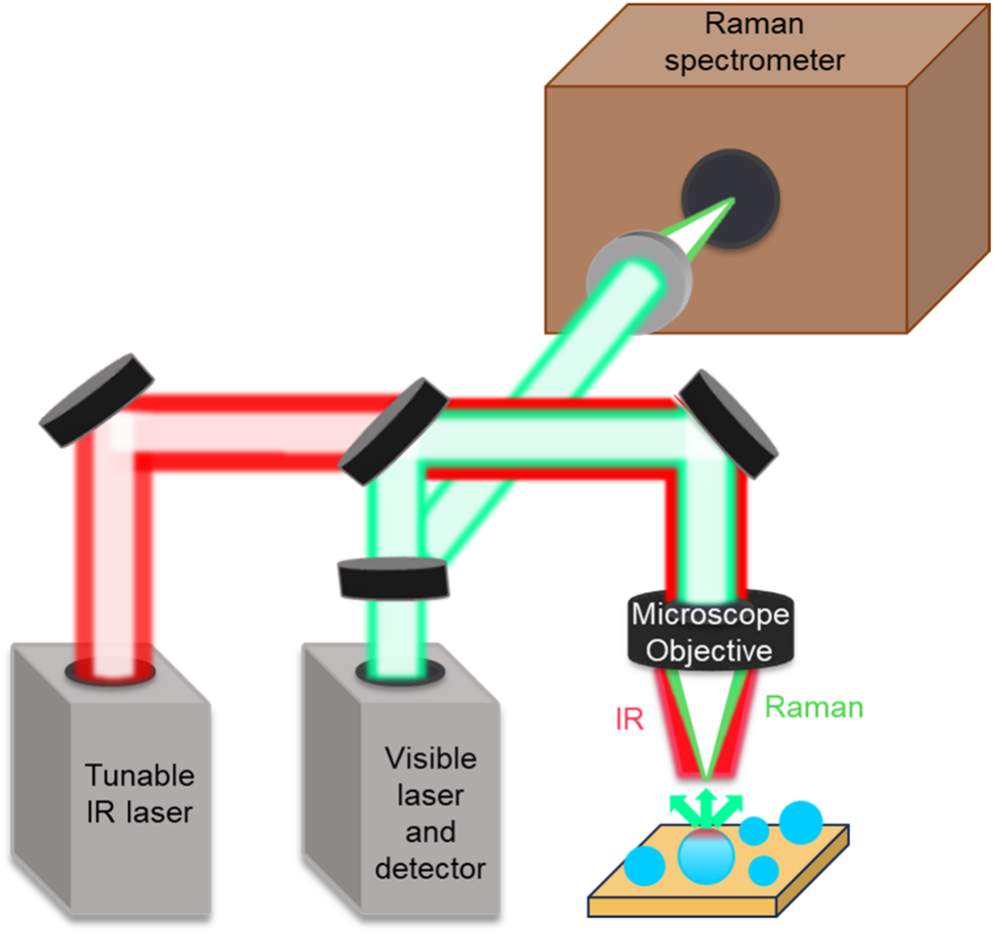
Simplified schematic representation of measurements of substrate-deposited single microdroplets. A tunable IR laser induces photothermal absorption in the sample, which is simultaneously probed by a coaligned visible laser through a microscope objective. The visible laser detects local refractive index changes, enabling collection of IR spectra from the spatial location. However, for these studies, O-PTIR spectroscopy was utilized to determine the presence of protonated fatty acids, deprotonated fatty acids and ester compounds, as discussed in Molina et al. (ref 50).

The microdroplets were deposited onto CaF_2_ substrates and then analyzed using the mIRage instrument between 10 to 30 min following droplet deposition on the substrate. During this time period, evaporation of volatile species (e.g., alcohol solvent and water byproduct) occurred. Spectra were recorded using the quantum cascade laser (QCL) that spans the 800–1950 cm^–1^ range to probe the fingerprint region. The particles were further examined using an optical parametric oscillator (OPO) laser covering the 2700–3600 cm^–1^ range, which primarily provided limited information related to the symmetric C–H stretching vibrations. Laser power was optimized for each sample to get the highest quality spectra with the IR laser power between 4 to 24% of the total power (100 mW) and the 532 nm laser power was varied between 2.4 to 40% of the total power (200 mW). The detector gain was set at 5× with an acquisition time of 1 min at 2 cm^–1^ spectral resolution. The detector gain was set at 5× with an acquisition time of 1 min at 2 cm^–1^ spectral resolution.

### Orbitrap High-Resolution Mass Spectrometry (HRMS)

Standard solutions of carboxylic acids prepared in methanol and ethanol were also analyzed using a direct-injection linear ion trap (ThermoFisher Orbitrap) HRMS. The molecules were ionized using an APCI source, which has been recognized as a more effective ionization technique for the analysis of esters in mass spectrometry.^52–54^ APCI ion source operates by vaporizing a continuous stream of aerosolized particles, allowing the resulting gas-phase molecules to undergo ionization. This ionization process occurs via a corona discharge generated between a high-voltage needle and the transfer capillary of the mass spectrometer, facilitating the production of charged analyte species in the gas phase.

HRMS analysis was conducted in positive ionization mode, yielding protonated molecular ions [M + H^+^]. The APCI ion source was operated at a temperature of 100 °C, with a capillary temperature of 300 °C. A discharge current of 5 μA was applied to facilitate ionization. The instrument was operated with a sheath gas flow rate of 10 arbitrary units and an auxiliary gas flow rate of 5 arbitrary units. The resolution was set to 240,000 FWHM. The mass spectrometry scan range was set to 50–400 Da to capture relevant analyte signals. The normalization level (NL), representing the ion counts per second, exceeded 1.00 E6 for nearly all samples. The elemental compositions corresponding to the observed *m*/*z* peaks were determined using the following atomic constraints: 12C (0–50), 1H (0–120), 16O (0–15) and 23Na (0–1). Peaks with mass tolerance of >4 ppm were rejected. Orbitrap mass spectra of carboxylic acids in methanol and their products (a) C_8_ carboxylic acid with a peak at *m*/*z* = 159.14, corresponding to methyl octanoate (MO + H^+^); (b) C_9_ carboxylic acid with a peak at *m*/*z* = 173.15, corresponding to methyl nonanoate (MN + H^+^); (c) C_12_ carboxylic acid with a peak at *m*/*z* = 215.20, corresponding to methyl laurate (ML + H^+^); (d) C_16_ carboxylic acid with a peak at *m*/*z* = 271.26, corresponding to methyl palmitate (MP + H^+^). Mass errors are given in Table 1 of Supporting Information (SI).

### Sample Preparation

For measurements of standards, including C_8_ acid, C_9_ acid, C_12_ acid, C_16_ acid, methyl octanoate, methyl nonanoate, methyl laurate, methyl palmitate, sodium octanoate, sodium nonanoate, sodium laurate, and sodium palmitate, were directly deposited onto calcium fluoride (CaF_2_) substrates, and the O-PTIR spectra were collected.

For measurements of reactions in microdroplets, O-PTIR spectra of single deposited microdroplets containing the ester product were collected. In these experiments, solid carboxylic acids, including C_12_ and C_16_ acids, were directly weighed into 20 mL scintillation vials with Teflon-lined caps, while liquid carboxylic acids were pipetted into separate vials. Three different alcohols were used as both a reactant and solvent and added to the vial to achieve a final concentration of 5 millimolal (mm). Microdroplets from each solution were formed utilizing a TSI 3076 atomizer, a Collison nebulizer, for 3 min while depositing the droplets onto CaF_2_ substrates for analysis with O-PTIR spectroscopy. The atomizer has a cyclone filter with a 40 μm element connected to it to remove microdroplets larger than 40 μm. However, there is the possibility of smaller droplets coalescing into bigger droplets.

The CaF_2_ substrate was placed in a Petri dish 1 to 2 cm away from the outlet of the atomizer. Following the 3 min deposition time, the Petri dish was closed and sealed with the Teflon tape for 10 to 30 min before position into the O-PTIR spectrometer. To prevent cross-contamination, the atomizer was thoroughly purged between samples using 60 mL of Milli-Q water followed by 20 mL of HPLC-grade methanol. Samples for direct-injection linear ion trap HRMS were prepared using the same procedure, with all glassware pretreated to eliminate trace contamination. The glass vials were combusted at 500 °C prior to use to ensure purity.

Solutions of C_9_ and C_12_ acids were also prepared with varying concentrations of water in methanol. The acid concentration was kept constant at 5 millimolal. For C_9_ acid, the amount of water varied to 0%, 1%, 1.5%, and 20% water by volume with respect to the volume of methanol. Similarly, for C_12_ acid, the amount of water varied to 0%, 0.5%, 1%, and 20% water by volume with respect to the volume of methanol. These solutions were atomized and analyzed through O-PTIR spectroscopy as described above.

### Hyperspectral Mapping of Individual Substrate-Deposited Microdroplets Utilizing O-PTIR Spectroscopy

Solutions were prepared and atomized as described previously. To collect spectra throughout the particle, a hyperspectral array feature was utilized on the mIRage software. The region of the deposited particles was selected, and an array of spectra was taken where every point was 1 μm apart from each other. The instrument first takes a spectrum of the top left of the selected region. Once finished with a single point, the spectrometer would translate to the next point, autofocus, and take another spectrum 1 μm away. This process was repeated in the array until the entire selected region had been analyzed. The data output is a matrix of spectra, where each set of (*x*, *y*) coordinates corresponds to a spectrum for that specific position. Peaks were deconvoluted in OriginLab, and a heatmap was produced using the coordinates and the integrated peak area.

## Results and Discussion

### O-PTIR Spectroscopy of Standard Compounds

O-PTIR spectroscopy is a highly suitable technique for distinguishing between various fatty acid species, such as carboxylic acids, carboxylate ions, and methyl esters, due to its ability to distinguish these species based on their carbonyl stretching frequencies.^50^ O-PTIR spectra of the different standards on CaF_2_ substrates were acquired for C_8_, C_9_, C_12_, and C_16_ carboxylic acids, their corresponding methyl esters, and sodium salts. The C_8_ and C_9_ compounds were in liquid form, while the C_12_ and C_16_ counterparts were solids (except methyl laurate, which was also a liquid at room temperature). Solid crystals were directly put on the CaF_2_ substrates for O-PTIR analysis. For liquid samples, a small aliquot was deposited onto the CaF_2_ substrate and subsequently analyzed using O-PTIR spectroscopy.

Figure 2 shows the O-PTIR spectra acquired for different standards. Panels (a), (b), and (c) display the spectra of sodium carboxylates, carboxylic acids, and methyl esters of carboxylic acids, respectively, across different chain lengths (C_8_, C_9_, C_14_, C_16_). Distinct spectral differences are observed among the different functional groups. Panel (a) displays a 1559 cm^–1^ vibrational band,^55^ characteristic of the asymmetric stretching mode of the deprotonated carboxylate (COO^–^) functional group. In panel (b), the characteristic C=O stretching vibration within the protonated carboxylic acid group at 1711 cm^–1^,^55^ confirms the presence of carboxylic acids, whereas panel (c) exhibits a C=O stretching band shifted to 1745 cm^–1^,^56,57^ corresponding to the ester functional group. The remaining vibrational bands correspond to other vibrational modes including, C–C, CH_2_ and CH_3_ modes as previously reported.^50^

**Figure 2:**
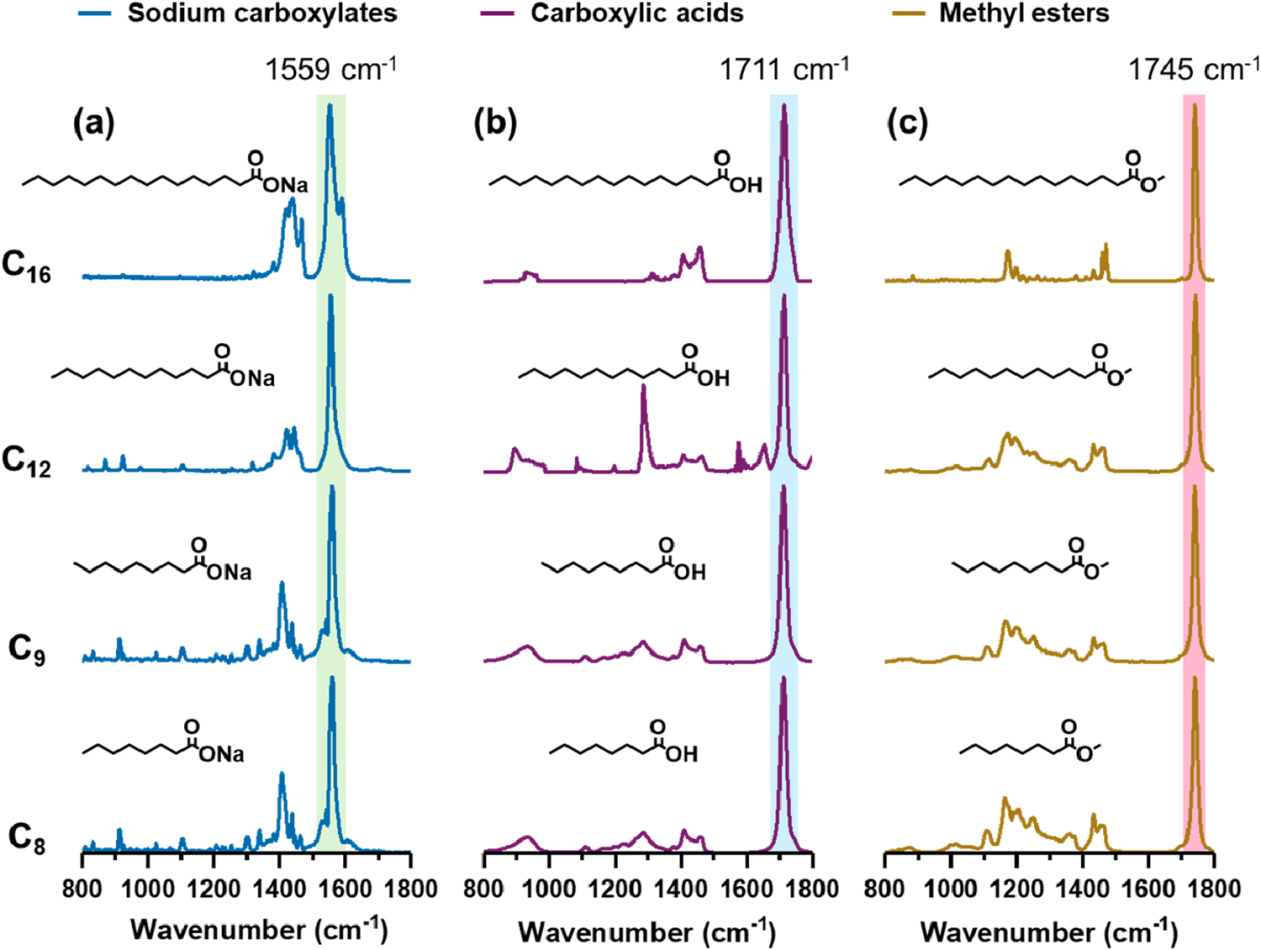
O-PTIR spectra of salts, acids, and esters of different chain lengths, C_8_, C_9_, C_12_, and C_16_, including (a) sodium carboxylates, characterized by the COO^–^ asymmetric stretching peak at 1559 cm^–1^; (b) carboxylic acids, identified by the C=O stretching peak at 1711 cm^–1^; and (c) methyl esters, distinguished by the C=O stretching peak at 1745 cm^–1^.

### O-PTIR Spectra of Deposited Microdroplets from C_8_ and C_9_ Carboxylic Acids Dissolved in Alcohol Solvents

Bulk solutions of carboxylic acids with varying chain lengths (C_8_, C_9_, C_12_ and C_16_) dissolved in methanol were analyzed using attenuated total reflection Fourier-transform infrared (ATR-FTIR) spectroscopy (Figure S1). All spectra exhibited a distinct band at 1711 cm^–1^, corresponding to the carbonyl stretching vibration of free carboxylic acids, with no evidence of ester-associated absorptions. To assess the effect of solvent evaporation in bulk solutions, the same solutions were dried onto an AMTIR crystal, and the resulting thin films were analyzed with ATR-FTIR spectroscopy (see Figure S2). Similarly, bulk solutions were also dried onto CaF_2_ substrates and analyzed with O-PTIR spectroscopy (see Figure S3). For all samples, both medium- and long-chain acids dissolved in alcohols, there was no evidence for esterification observed, confirming that esterification does not occur either in bulk alcohol solutions or upon drying bulk solutions.

Solutions of C_8_ and C_9_ acids were prepared at a concentration of 5 millimolal in methanol, ethanol, and IPA. The solutions were sent through a Collison nebulizer to form microdroplets, which were subsequently deposited onto CaF_2_ substrates for O-PTIR analysis, as discussed in the Materials and Methods Section. This produced microdroplets that ranged in size from 2 to 20 μm after drying. The O-PTIR spectra of the deposited and dried microdroplets are presented in Figure 3.

**Figure 3:**
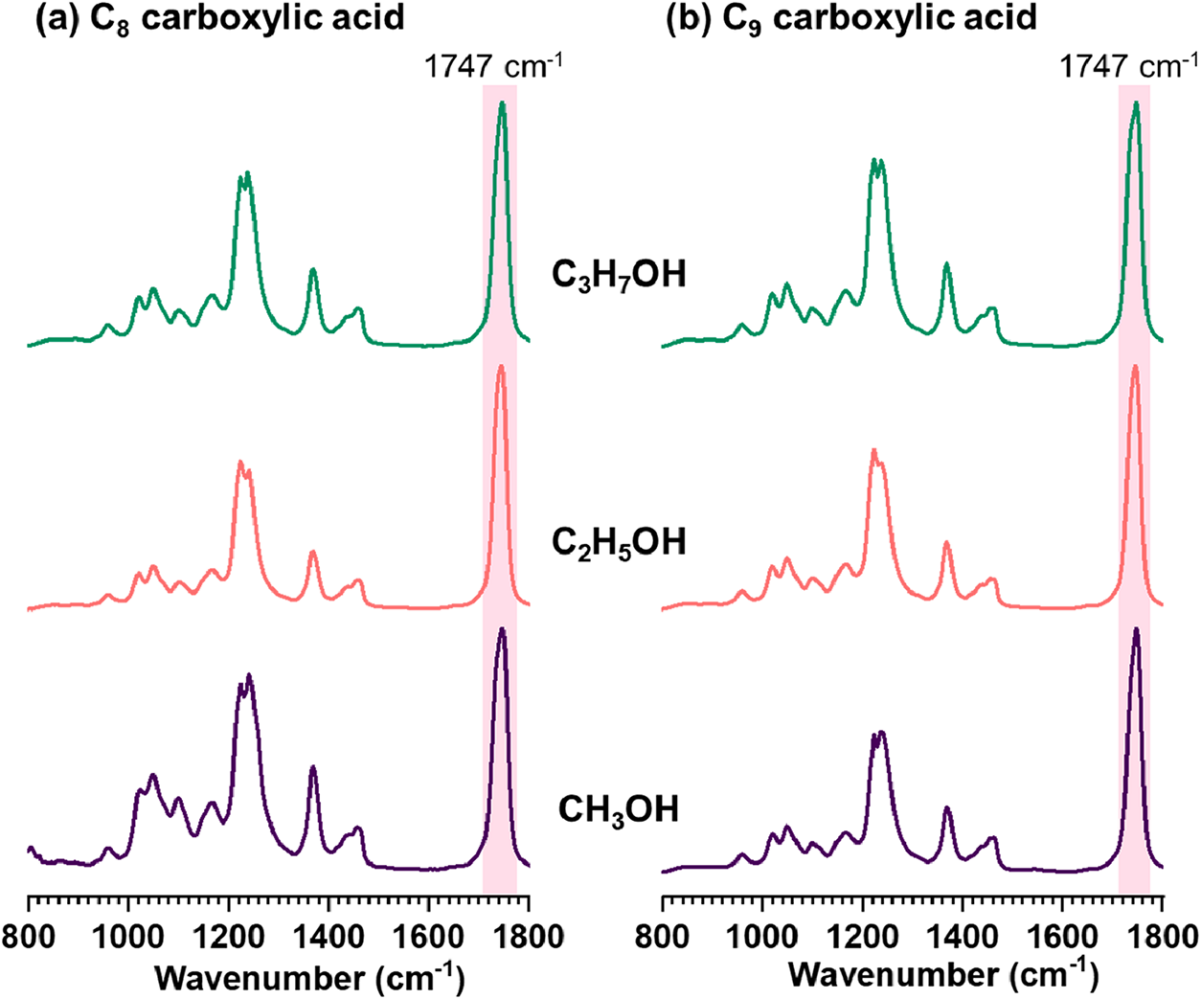
O-PTIR spectra of microdroplets deposited and dried onto CaF_2_ substrates following reaction of carboxylic acids dissolved in alcohol solutions: (a) C_8_ and (b) C_9_ carboxylic acids in methanol (C_1_), ethanol (C_2_), and isopropyl alcohol (C_3_). The highlighted region at 1747 cm^–1^ corresponds to the C=O stretching vibration consistent with the ester and indicating the occurrence of esterification reactions at the microdroplet–air interface.

The O-PTIR spectra of deposited droplets from both C_8_ and C_9_ carboxylic acids in alcohols exhibit a vibrational band at 1747 cm^–1^, characteristic of the C=O ester stretching mode. There is no evidence of a band around 1711 cm^–1^ associated with the C=O stretching of carboxylic acid. Our results show that the esterification is taking place from the microdroplets formed from a bulk solution of carboxylic acid in alcohol. One possible explanation is that esterification may be promoted at the microdroplet interface, where the increased surface area-to-volume ratio could facilitate greater interaction between carboxylic acid and alcohol. As medium-chain carboxylic acids, C_8_ and C_9_ exhibit higher solubility in alcohol, allowing them to remain well-dissolved in the bulk solution. During the process of forming the microdroplet, the evaporation of alcohol results in the enrichment of carboxylic acid molecules within the microdroplet and at the microdroplet interface. This increases the local acidity at the interface leading to the esterification at the interface. Additionally, once esterification occurs within the microdroplet, the water product can readily evaporate from the microdroplet into the gas phase. This continuous removal of water effectively shifts the equilibrium toward product formation.

The OPO laser was also employed to probe the C–H stretching region. As shown in Figure S4, microdroplets produced from C_8_ and C_9_ carboxylic acids in methanol exhibit absorption bands at 2856 and 2928 cm^–1^, characteristic of typical C–H stretching vibrations for long-chain aliphatic hydrocarbons.

### O-PTIR Spectra of Deposited Microdroplets from C_12_ and C_16_ Carboxylic Acids Dissolved in Alcohol Solvents

Solutions of C_12_ and C_16_ acids were similarly prepared at a concentration of 5 millimolal in methanol, ethanol, and IPA and then microdroplets were formed and deposited onto CaF_2_ substrates for O-PTIR analysis. The O-PTIR spectra of the aerosolized particles are presented in Figure 4.

**Figure 4:**
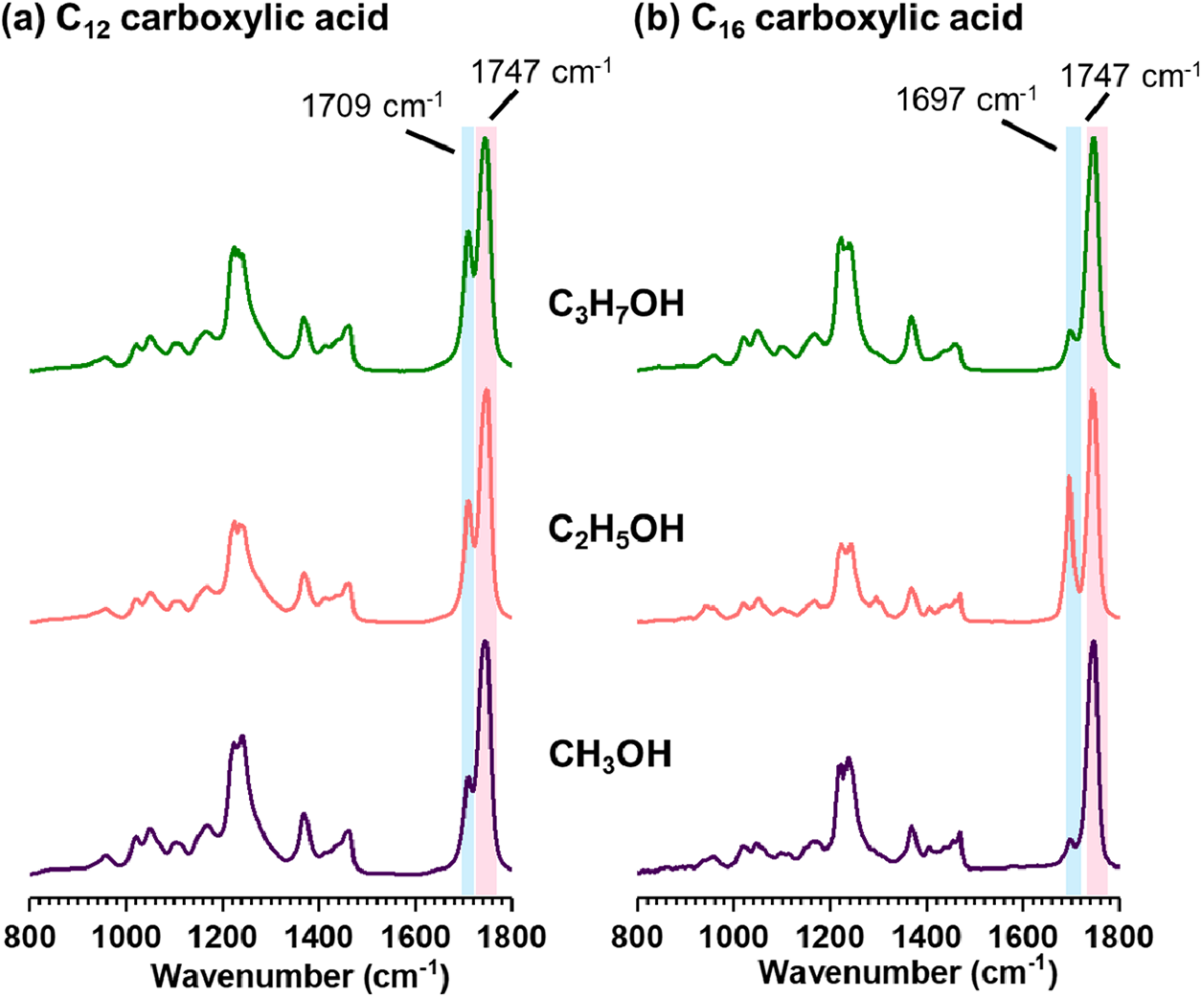
O-PTIR spectra of aerosolized droplets deposited and dried onto CaF_2_ substrates formed from carboxylic acids in alcohol: (a) C_12_ and (b) C_16_ carboxylic acids in methanol (C_1_), ethanol (C_2_), and isopropyl alcohol (C_3_). The highlighted region shows the presence of both the C=O stretching vibration, with the peak at 1747 cm^–1^ attributed to the ester C=O stretch, and the peaks at 1697 cm^–1^ and 1709 cm^–1^ corresponding to the C=O stretch of carboxylic acids.

For deposited microdroplets of longer-chain carboxylic acids in alcohols, such as C_12_ and C_16_, the O-PTIR spectra exhibit two distinct absorption peaks. The first, observed at 1747 cm^–1^, corresponds to the C=O stretching vibration of an ester, indicating that esterification occurs similarly to that of medium-chain fatty acids. The second peak appears near 1700 cm^–1^ (specifically, 1709 cm^–1^ for C_12_ and 1697 cm^–1^ for C_16_) and is likely attributed to a shifted C=O stretching vibration of a hydrogen-bonded dimer of a carboxylic acid.^58^ As long-chain carboxylic acids, C_12_ and C_16_ exhibit limited solubility in alcohol, resulting in incomplete dissolution within the bulk phase. In combination with our findings, this suggests that during microdroplet formation, a fraction of carboxylic acid molecules at the microdroplet interface undergo esterification; however, the remaining carboxylic acid molecules do not. One plausible explanation is that hydrogen bonding between carboxylic acids occurred, resulting in dimer formation, which subsequently limited esterification for these longer-chain fatty acids. Max et al.^58^ showed that hydrogen-bonded carboxylic acids exhibit characteristic C=O stretching vibrations at 1688 cm^–1^ and 1700 cm^–1^, supporting the presence of dimers in these samples.

### Orbitrap High-Resolution Mass Spectra of Carboxylic Acids in Methanol and Ethanol

Solutions of C_8_, C_9_, C_12_, and C_16_ carboxylic acids were prepared in methanol and ethanol and analyzed using Orbitrap HRMS. The APCI method was employed to ionize the particles, which were subsequently detected and analyzed based on their specific mass-to-charge (*m*/*z*) ratios.

Figures 5 and 6 display the mass spectra of C_8_, C_9_, C_12_, and C_16_ carboxylic acids in methanol and ethanol, respectively. Figure 5(a) presents two distinct *m*/*z* peaks at 145.12 and 159.14 for standard C_8_ acid in methanol. As discussed in Figure S5­(a), the *m*/*z* peak at 145.12 corresponds to standard C_8_ acid. The additional peak at *m*/*z* = 159.15 represents an increment of *m*/*z* = 14, likely resulting from the addition of a −CH_2_ group. This shift modifies the molecular formula of C_8_ acid (C_8_H_16_O_2_) to C_9_H_18_O_2_, which corresponds to methyl octanoate, a methyl ester. These findings demonstrate that the process of aerosolizing C_8_ acid in methanol using the APCI source in mass spectrometry leads to esterification. Given the large differences in these methods and the time scales of these experiments, the HRMS experiment was used for one purpose: to confirm, through mass analysis, the ester product, rather than for a quantitative comparison of product yield.

**Figure 5:**
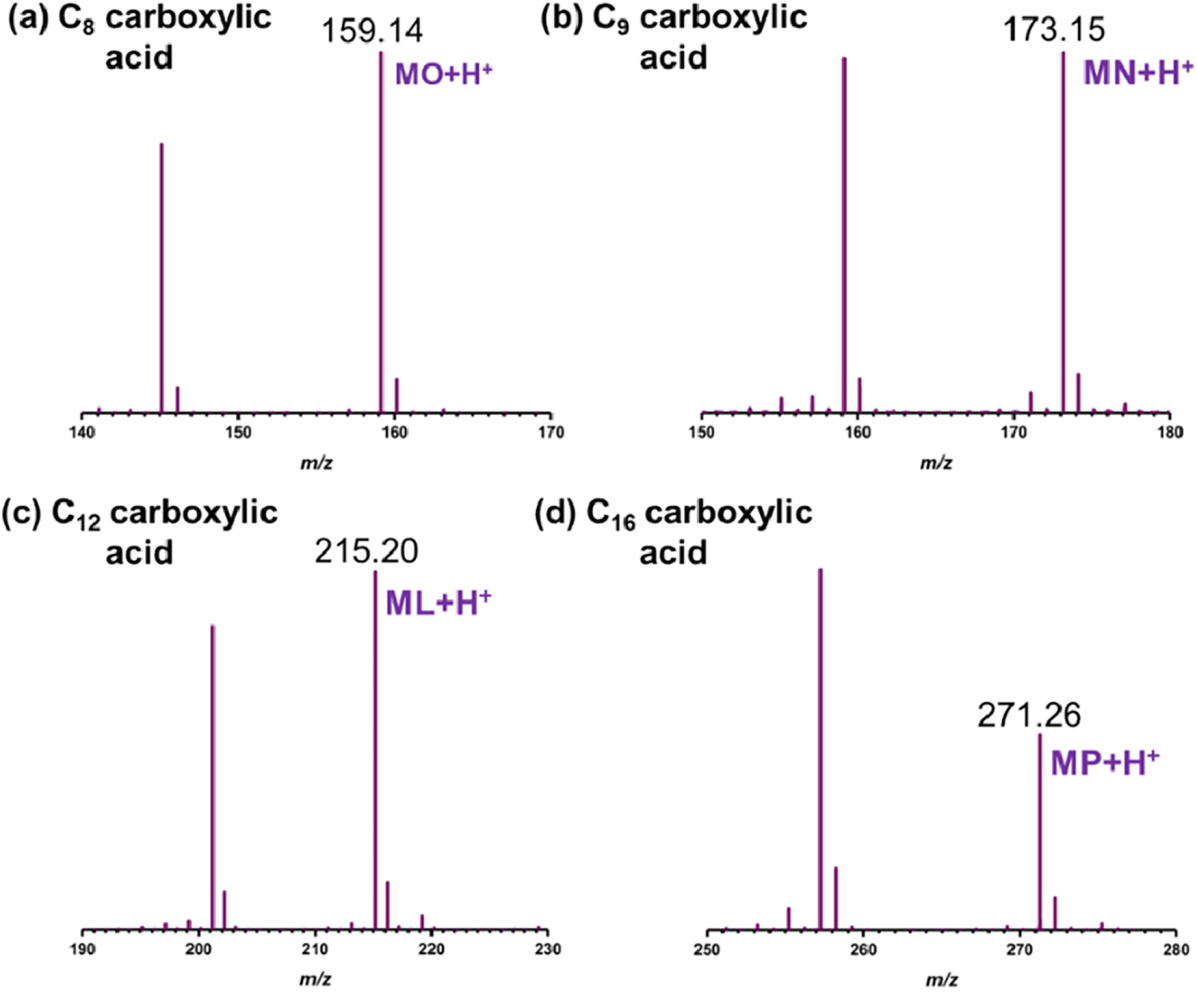
Orbitrap mass spectra of carboxylic acids in methanol: (a) C_8_ carboxylic acid with a peak at *m*/*z* = 159.14, corresponding to methyl octanoate (MO + H^+^); (b) C_9_ carboxylic acid with a peak at *m*/*z* = 173.15, corresponding to methyl nonanoate (MN + H^+^); (c) C_12_ carboxylic acid with a peak at *m*/*z* = 215.20, corresponding to methyl laurate (ML + H^+^); (d) C_16_ carboxylic acid with a peak at *m*/*z* = 271.26, corresponding to methyl palmitate (MP + H^+^).

**Figure 6:**
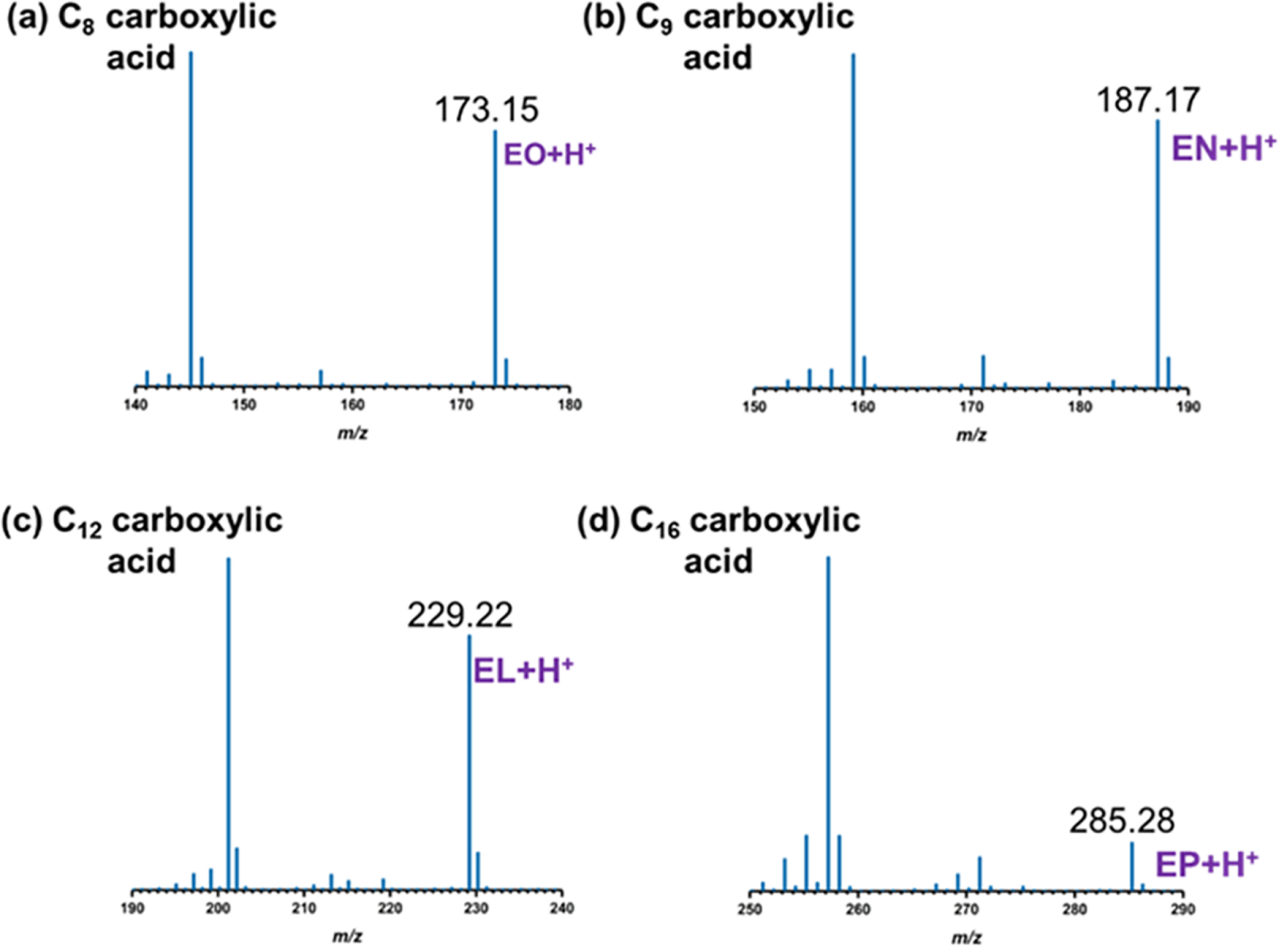
Orbitrap mass spectra of carboxylic acids in ethanol: (a) C_8_ carboxylic acid with a peak at *m*/*z* = 173.15, corresponding to ethyl octanoate (EO + H^+^); (b) C_9_ carboxylic acid with a peak at *m*/*z* = 187.17, corresponding to ethyl nonanoate (EN + H^+^); (c) C_12_ carboxylic acid with a peak at *m*/*z* = 229.22, corresponding to ethyl laurate (EL + H^+^); (d) C_16_ carboxylic acid with a peak at *m*/*z* = 285.28, corresponding to ethyl palmitate (EP + H^+^).

Similarly, Figure S5­(b–d), display two distinct peaks for standard C_9_, C_12_, and C_16_ carboxylic acids in methanol. For C_9_, the observed peaks at *m*/*z* = 159.14 correspond to C_9_ acid (C_9_H_18_O_2_), while the peak at *m*/*z* = 173.15 is attributed to methyl nonanoate. In the case of C_12_, the spectrum exhibits a peak at *m*/*z* = 201.19, corresponding to C_12_ acid (C_12_H_24_O_2_), and another at *m*/*z* = 215.20, associated with methyl laurate. Similarly, for C_16_, the peaks at *m*/*z* = 257.25 and 271.26 correspond to C_16_ acid (C_16_H_32_O_2_) and methyl palmitate, respectively. These results indicate that esterification occurs in microdroplets containing these carboxylic acids in methanol, leading to the formation of their respective methyl esters.

Figure 6 shows that the results observed for carboxylic acids in ethanol were consistent with methanol, also exhibiting two distinct *m*/*z* peaks, when analyzed using HRMS. For the C_8_ carboxylic acid, a peak at *m*/*z* 145.12 corresponded to C_8_ acid (C_8_H_16_O_2_), as shown in Figure S5­(a). Additionally, a peak at *m*/*z* 173.15 displayed a mass difference of 28.03, likely indicating the incorporation of a C_2_H_4_ moiety, corresponding to ethyl octanoate (C_10_H_20_O_2_). A similar pattern was observed for the C_9_ carboxylic acid, with a peak at *m*/*z* 159.14 assigned to C_9_ acid and a corresponding peak at *m*/*z* 187.17 attributed to ethyl nonanoate. For the C_12_ carboxylic acid, *m*/*z* 201.19 corresponded to C_12_ acid, while *m*/*z* 229.22 was assigned to ethyl laurate. In the case of C_16_, *m*/*z* 257.25 was identified as C_16_ acid, with an additional peak at *m*/*z* 285.28 indicative of ethyl palmitate.

APCI is a soft ionization technique that utilizes a corona discharge to ionize molecules, primarily through proton transfer mechanisms. Solvents such as methanol and ethanol can undergo protonation, forming protonated species (CH_3_OH_2_^+^ and C_2_H_5_OH_2_^+^, respectively), which act as protic catalysts that facilitate surface chemistry and interfacial reactions during ionization. Additionally, when carboxylic acids are aerosolized into the APCI ionization region, the evaporation of alcohol leads to an increased acid concentration at the microdroplet interface. This results in a localized acidic microenvironment, which can accelerate esterification by promoting proton transfer and enhanced reactivity at the droplet surface. Although the mass spectra show both the acid and ester for medium-chain fatty acids, which differs from the O-PTIR spectra that show only esters in the residual microdroplet particles, the mass spectra are collected within seconds of aerosolization, whereas the time scales for the single particle methods are 10–30 min. It is these longer time scales that are most interesting in this study, as it shows that esterification is quantitative for deposited microdroplets.

### Effects of Water on the Esterification Reaction

To investigate the effect of water on the microdroplet esterification reaction, solutions of C_8_, C_9_, C_12_, and C_16_ carboxylic acids were prepared in a solvent mixture of methanol and water at an 80:20 volume ratio. These solutions were used to form substrate-deposited microdroplets, which were analyzed using O-PTIR spectroscopy. Notably, the spectral data revealed very different chemistry occurring, as evident by the O-PTIR spectra for particles, as shown in Figure 7.

**Figure 7:**
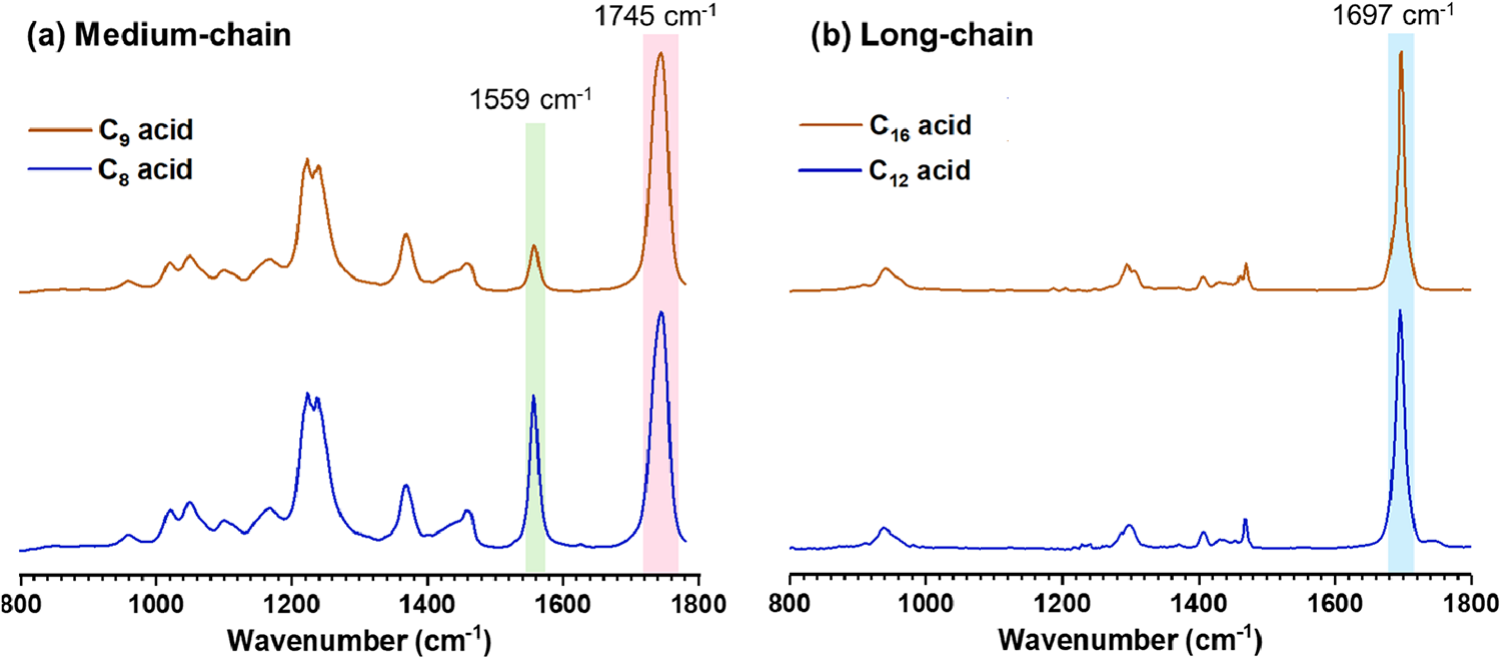
O-PTIR spectra of aerosolized droplets deposited and dried onto CaF_2_ substrates formed from carboxylic acids in methanol–water mixture (80:20 volume ratio). (a) Medium-chain carboxylic acids show peaks at 1745 cm^–1^ and 1559 cm^–1^ indicating ester formation via esterification and carboxylate ion formation due to dissolution in water for C_8_ and C_9_ carboxylic acids. (b) Long-chain carboxylic acids show peak at 1697 cm^–1^ suggesting no esterification for C_12_ and C_16_ carboxylic acids.

In particular, Figure 7(a) presents O-PTIR spectra for C_8_ and C_9_ carboxylic acids, revealing a dominant peak at 1745 cm^–1^, characteristic of the C=O stretching vibration of an ester, as discussed in the previous section. Additionally, a new peak at 1559 cm^–1^ is detected. This corresponds to the carboxylate asymmetric stretching mode of the carboxylate (ν_a_(COO^–^)) ion, i.e., the deprotonation of the medium-chain carboxylic acid. Medium-chain carboxylic acids, C_8_ and C_9_, exhibit some solubility in both alcohol and water, allowing for effective dispersion within this water/alcohol bulk phase. Under these conditions, the carboxylic acid molecules may undergo esterification or proton dissociation, facilitated by their dissolution in the water initially present upon microdroplet formation.

Figure 7(b) presents O-PTIR spectra for C_12_ and C_16_ carboxylic acids reacting in alcohol/water mixtures, showing no detectable peak around 1745 cm^–1^. This absence suggests that the presence of water inhibits the esterification reaction. As long-chain carboxylic acids, C_12_ and C_16_ exhibit lower solubility compared to their shorter-chain counterparts, C_8_ and C_9_. At the interface, we propose that these molecules engage in hydrogen bonding with other carboxylic acid molecules, leading to dimer formation. Consequently, the availability of free carboxylic acid molecules for esterification is significantly reduced, ultimately inhibiting esterification. This results in the predominant observation of C=O stretch at 1697 cm^–1^, characteristic of hydrogen-bonded carboxylic acid dimers.

To investigate the effect of water content on the speciation of medium- and long-chain carboxylic acids in methanol, we analyzed how the relative contributions of carbonyl species change with increasing water volume percentage. Figure 8 displays O-PTIR spectra of C_9_ acid (Figure 8(a)) and C_12_ acid (Figure 8(b)) in methanol with water concentrations ranging from 0 to 20% by volume.

**Figure 8:**
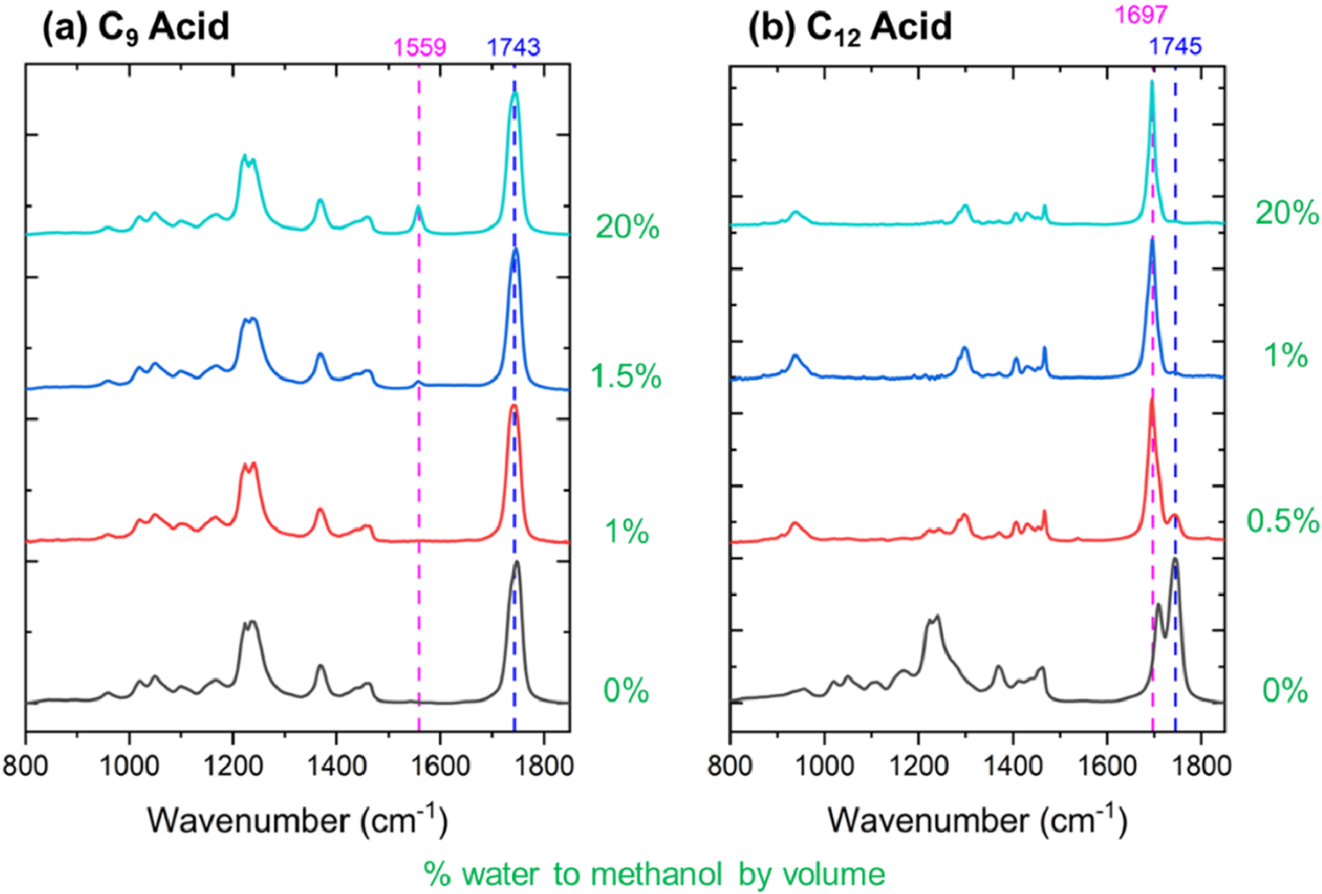
O-PTIR spectra of microdroplets containing carboxylic acids deposited onto CaF_2_ substrates. Droplets were generated from water–methanol mixtures with varying water volume percentages (in green): (a) C_9_ acid; (b) C_12_ acid. The highlighted spectral regions correspond to C=O stretching vibrations associated with distinct species: 1559 cm^–1^ (carboxylate anion), 1697 cm^–1^ (carboxylic acid dimer), and 1743 or 1745 cm^–1^ (ester).

For C_9_ acid (Figure 8(a)), the ester peak at 1743 cm^–1^ dominates at low water concentrations (≤1%), with minimal evidence of carboxylate formation. A clear emergence of the asymmetric carboxylate stretch at 1559 cm^–1^ is observed at ≥1.5% water, suggesting sufficient ionization of the acid into its deprotonated form. This transition likely reflects increased solvation and stabilization of the carboxylate anion in a more water-solvated environment.

In contrast, the O-PTIR spectra of C_12_ acid (Figure 8(b)) reveal insights into the impact of water on the reaction chemistry. At 1% water and above, the carboxylic acid appears predominantly dimerized, as evidenced by the persistent shift near 1697 cm^–1^ indicative of hydrogen-bonded carboxylic acid dimers. Below 1% water, both ester and dimer features are present, implying that at low hydration levels, the acid is not fully solvated, and some esterification or aggregation occurs. The data suggest that longer-chain acids, such as C_12_ acid, preferentially form dimers at the microdroplet interface, while medium-chain acids, such as C_9_ acid, more readily deprotonate with modest increases in water content.

### Spatial Distribution and Colocalization of Carbonyl Species in Microdroplets via Hyperspectral O-PTIR Imaging

To investigate any spatial distribution of carbonyl-containing species within individual microdroplets, we examined the localization of ester and carboxylate functional groups in C_9_ acid droplets formed from a methanol–water mixture (80:20 v/v).

For C_9_ acid in methanol–water, hyperspectral O-PTIR imaging, as shown in Figure 9, was used to generate spatial heatmaps of the C=O stretching vibrations corresponding to the ester (1743 cm^–1^) and the carboxylate anion (1559 cm^–1^). These heatmaps reveal the distinct spatial localization of each species. The overlay in Figure 9(d) highlights heterogeneous regions within the particle, with some domains exhibiting elevated ester signal and others enriched in carboxylate, suggesting partial phase separation of these species upon microdroplet drying. We also calculated the spatial distribution of carboxylate ion content as a percentage of total carbonyl signal, using the ratio of the carboxylate peak area to the sum of carboxylate and ester peak area at each pixel. The resulting map shows differences observed for the ratio of the % 1559 cm^–1^ peak. The percentage increases from 11.6% to 67.9% within the particle, suggesting that some phase separation between ester and carboxylate species is occurring in the deposited microdroplet particle. A lower percentage value of 11.6% (blue regions in the map) corresponds to a lower contribution of the carboxylate signal compared to the ester signal at those pixels, whereas a higher value of 67.9% (red regions) indicates an increase in the relative abundance of carboxylate species with respect to the ester.

**Figure 9:**
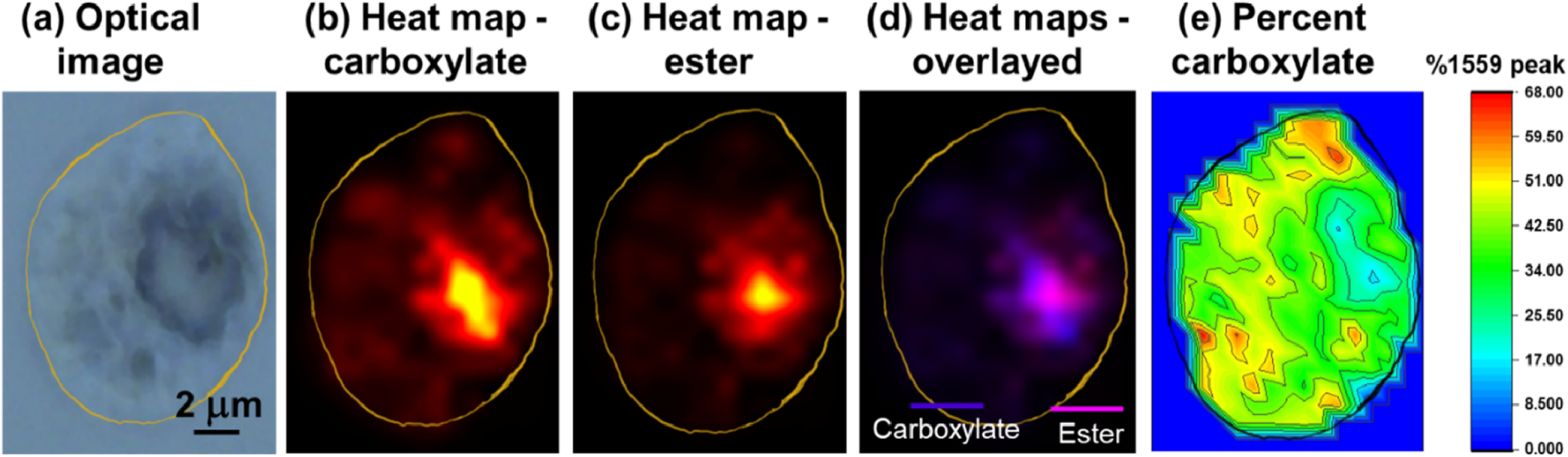
Spatially resolved O-PTIR analysis of a substrate-deposited microdroplet formed from C_9_ acid in methanol:water (80:20 by volume). (a) Optical image of the substrate-deposited microdroplet residue deposited on a CaF_2_ substrate. (b) Heatmap of the integrated C=O asymmetric stretching band corresponding to the carboxylate anion. (c) Heatmap of the integrated ester C=O stretching band. (d) Color map showing the spatial distribution of functional groups within a microdroplet. Blue indicates regions enriched in carboxylate, while pink highlights the presence of ester functionalities. (e) Spatial distribution of carboxylate ion signal expressed as the percentage of carboxylate ion signal to ester signal, calculated by normalizing the carboxylate peak area to the ester peak area at each pixel. This shows there are some differences observed as the percentage of the 1559 cm^–1^ peak signal increases from 11.6% to 67.9% within the particle when normalized to the ester peak signal. This percentage change demonstrates that the microdroplet is not uniform in composition.

## Conclusion

Esterification is a widely utilized reaction in industrial processes, typically requiring a catalyst to drive the reaction equilibrium forward. This study investigates esterification reactions within alcohol microdroplets to facilitate reactions occurring at the interface for fatty acids of varying chain lengths, providing additional insights into the role of interfacial esterification reactions. Previous research has demonstrated the potential of microbubble-mediated esterification, where oleic acid reacts with methanol vapors. The small size and high interfacial area of microbubbles enhance mass transfer at the bubble interface, facilitating equilibrium shift and increasing the esterification rate.^47^ Our findings here from O-PTIR spectroscopy of individual microdroplets deposited onto a substrate, along with mass spectrometry, reveal that microdroplets containing medium-chain carboxylic acids (e.g., C_8_ and C_9_) undergo complete esterification in methanol, ethanol, and IPA. In contrast, microdroplets of longer-chain carboxylic acids (e.g., C_12_ and C_16_) do not favor complete esterification under the same conditions. Additionally, we show that microdroplets of medium-chain acids in a methanol–water mixture exhibit both esterification and deprotonation at the microdroplet interface. However, for long-chain carboxylic acids, these surface reactions are completely inhibited. These findings suggest that esterification can occur on the microdroplet interface during their formation.

Figure 10 summarizes the esterification behavior of medium- and long-chain carboxylic acids as probed by infrared spectroscopy, O-PTIR and ATR-FTIR, respectively. Figure 10(a) shows that for medium-chain acids in methanol, no esterification was detected in bulk solutions, regardless of the presence of water. Upon formation of the microdroplets, esterification occurred at the microdroplet interface in the absence of water, whereas the presence of water suppressed ester formation due to enhanced deprotonation and stabilization of the carboxylate anion. Figure 10(b) shows that for long-chain acids in methanol, no esterification was observed in bulk solutions with or without water. Partial esterification was detected in the absence of water for microdroplets, and esterification was completely inhibited in the presence of water.

**Figure 10:**
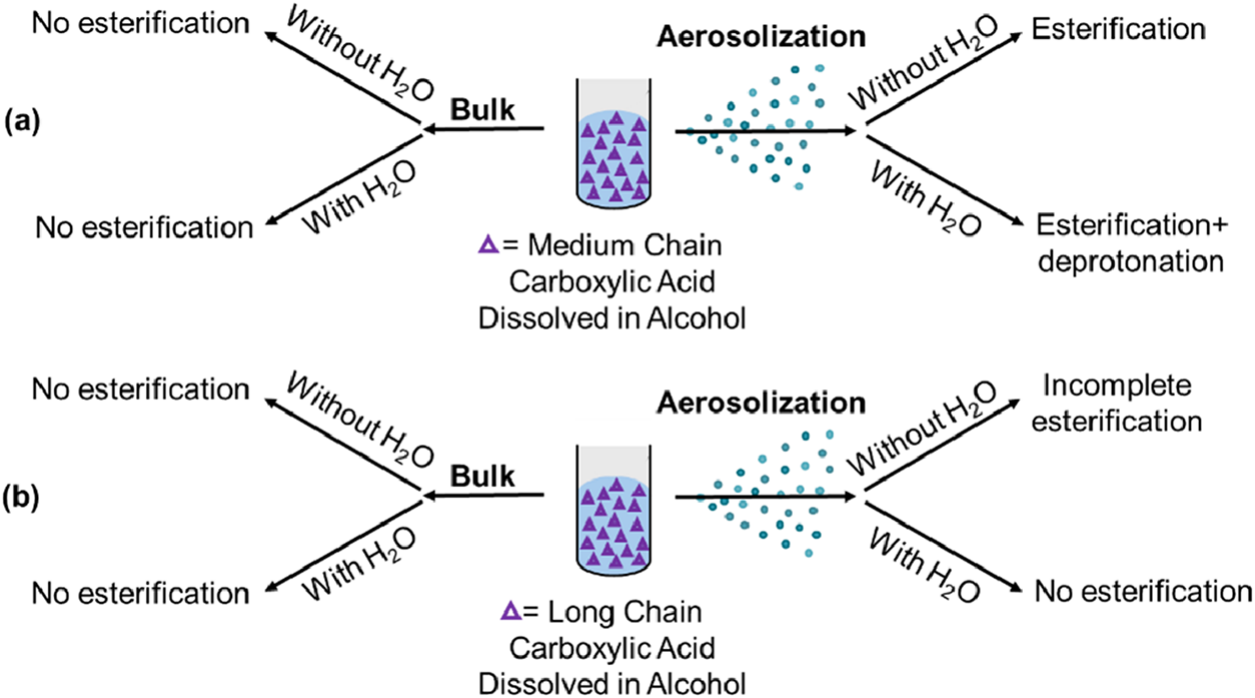
Summary of experimental results comparing carboxylic acid systems. (a) Medium-chain carboxylic acids examined in bulk and microdroplet phases under dry and aqueous conditions. (b) Long-chain carboxylic acids examined in bulk and aerosolized phases under dry and aqueous conditions.

Hyperspectral O-PTIR imaging was employed to assess the spatial distribution of carbonyl-containing species within individual droplets. Although there was some mixing of medium-chain fatty acids, the ester and carboxylate functionalities were found to be colocalized throughout the particle, however, the percentage of these varied within the particle suggesting some phase separation.

Overall, our findings, based on O-PTIR and HRMS analyses, demonstrate that the esterification reaction between carboxylic acids when dissolved in alcohols occurs for microdroplets, reactions that are not observed in bulk solutions or upon evaporation of bulk solutions. The extent of esterification is influenced by the chain length of the carboxylic acid, with shorter chains favoring higher reactivity. Additionally, our results reveal that the presence of water inhibits or completely suppresses the esterification reaction through two different mechanisms, deprotonation of the carboxylic acid for medium-chain fatty acids and hydrogen-bonded dimerization for longer-chain fatty acids. This study highlights the potential to consider this as an environmentally sustainable approach for esterification reactions, eliminating the need for heat or catalysts. Overall, this method holds some potential in enabling esterification and advancing novel chemical processing, as well as providing an additional example of enhanced reactivity within microdroplets relative to bulk solutions.

## Supplementary Material



## Notes

The Supporting Information (SI) contains additional information on experimental methods, 5 figures, which include bulk ATR-FTIR spectra of C_8_, C_9_, C_12_ and C_16_ carboxylic acids in methanol solvent anddried onto AMTIR crystal, O-PTIR spectra of C_8_, C_9_, C_12_ and C_16_ carboxylic acids in methanol dried onto CaF_2_ substrate, O-PTIR spectra of C_8_, C_9_, C_12_ and C_16_ carboxylic acids in methanol solvent, an Orbitrap HRMS of C_8_, C_9_, C_12_, and C_16_ carboxylic acids in acetonitrile solvent, and 1 table with m/z values and mass errors for esterification products.(PDF)
