# Supplementary material for: Ester Formation in Alcohol Microdroplet Sprays: Enhanced Reactivity of C8 to C16 Carboxylic Acids with C1 to C3 Alcohols and the Effect of Water
Source: J Phys Chem A. 2025 Nov 17;129(47):10807–18. doi: 10.1021/acs.jpca.5c04566 (PMC12670510; doi:10.1021/acs.jpca.5c04566)
Supplement: Supplementary file 1 [file jp5c04566_si_001.pdf]

Supporting Information for

Ester Formation in Alcohol Microdroplet Sprays: Enhanced Reactivity of C_8 to C_{16} Carboxylic Acids with C_1 to C_3 Alcohols and the Effect of Water

Lincoln Mehndiratta, Justin Wang, Jonathan H. Slade, Vicki H. Grassian*

Department of Chemistry & Biochemistry, University of California San Diego, 9500 Gilman Dr, CA. 92037

*Correspondence should be addressed to Vicki H. Grassian (vhgrassian@ucsd.edu)

The Supporting Information (SI) contains additional information on the experimental methods as well as five figures and one table.

Additional Information on Experimental Methods

Attenuated total reflection Fourier transform infrared (ATR-FTIR) Spectroscopy. ATR-FTIR spectroscopy was employed to measure the infrared spectra of C_8, C_9, C_{12} and C_{16} carboxylic acids in methanol. Additionally, these solutions were also dried onto the AMTIR crystal and dried film spectra were also obtained. Spectral acquisition was performed using a Nicolet iS10 FTIR spectrometer (Thermo Fisher Scientific) equipped with a mercury cadmium telluride (MCT/A) detector and an Amorphous Material Transmitting Infrared Radiation (AMTIR) crystal housed within a Teflon-coated flow cell (PIKE Technologies), as described previously. [1,2] Solutions of C_8 and C_9 were made at 1 mg/mL concentration in methanol to deposit on the AMTIR crystal. Solutions of C_{12} and C_{16} acids were prepared at a concentration of 20 mg/mL in methanol and sonicated to ensure homogeneity prior to deposition on the AMTIR crystal for 5 -10 minutes. Infrared spectra were recorded using 100 scans over the range spectral range from 1200 to 1800 cm^{-1} at a spectral resolution of 4 cm^{-1}. Spectra were baseline-corrected and processed using OMNIC software and plotted using Origin software.

Infrared spectra of C_8, C_9, C_{12} and C_{16} acids dissolved in methanol and dried. Figure S1 shows ATR-FTIR spectra of bulk C_8, C_9, C_{12}, and C_{16} carboxylic acids in methanol measured using an AMTIR crystal placed in a horizontal cell (Pike Technologies). All spectra show the characteristic C=O stretching band at 1711 cm^{-1}, for carboxylic acids, with no evidence of ester formation at 1745 cm^{-1}, indicating that esterification is not observed in bulk solution at room temperature which is well known that catalysts or heating is needed (see discussion in main text). Figure S2 shows ATR-FTIR spectra of the same carboxylic acids in methanol after drying onto the AMTIR crystal. In Figure S2 (a), the medium-chain acids retain the free acid C=O stretch at 1711 cm^{-1}, again confirming the absence of esterification. In contrast, Figure S2 (b) shows that the long-chain acids display a shifted C=O band at 1698 cm^{-1}, consistent with carboxylic acid dimer formation. Similar

experiments were done for the drying of bulk solutions of carboxylic acids in methanol on a CaF_2 substrate and analyzing with O-PTIR spectroscopy (Figure S3). Most importantly is the fact that there is no evidence of ester formation observed for dried bulk solutions. Together, all of these results confirm that esterification does not occur either in the bulk or through drying of bulk solutions containing carboxylic acids in methanol.

O-PTIR spectra of microdroplets in the C–H stretching region. Microdroplets formed from C_8, C_9, C_{12}, and C_{16} carboxylic acids dissolved in methanol and then deposited on a CaF_2 substrate were analyzed using O-PTIR spectroscopy with an OPO laser to probe the C-H stretching region. All four acids exhibited characteristic absorption bands at 2856 and 2928 cm^{-1}, corresponding to C–H stretching vibrations, as shown in Figure S4.

Supporting Information Figures

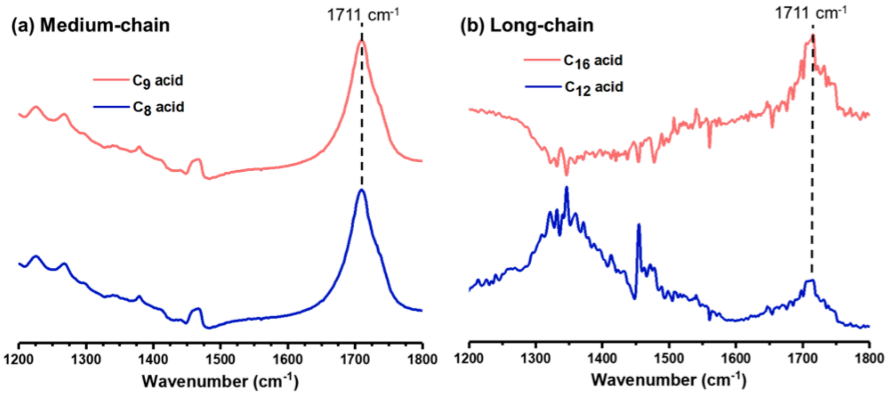

Figure S1. ATR-FTIR spectra of bulk (a) medium-chain carboxylic acids and (b) long-chain carboxylic acids in methanol, exhibiting a C=O stretching vibration at 1711 cm^{-1}, indicative of the carboxylic acid functional group.

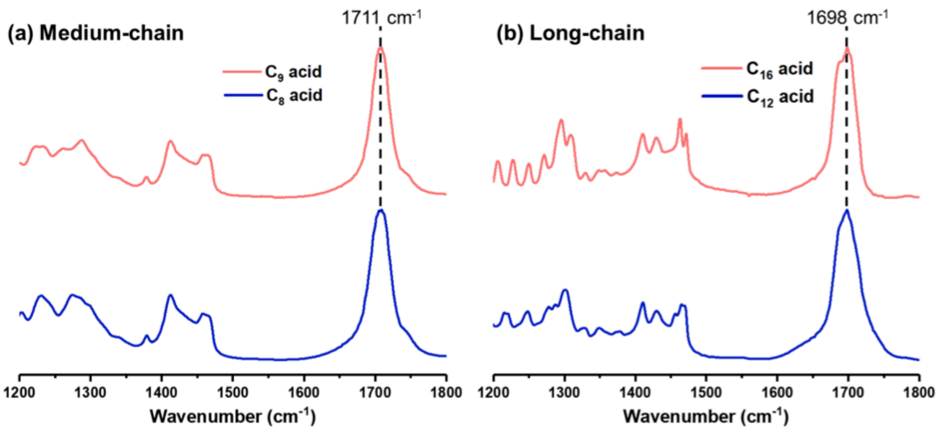

Figure S2. ATR-FTIR spectra of (a) medium-chain carboxylic acids and (b) long-chain carboxylic acids in methanol dried onto the AMTIR crystal. Medium-chain exhibits a C=O stretching vibration at 1711 cm^{-1}, indicative of the carboxylic acid functional group while long-chain exhibiting C=O stretching vibration at 1698 cm^{-1} from hydrogen bonded carboxylic acid dimer.

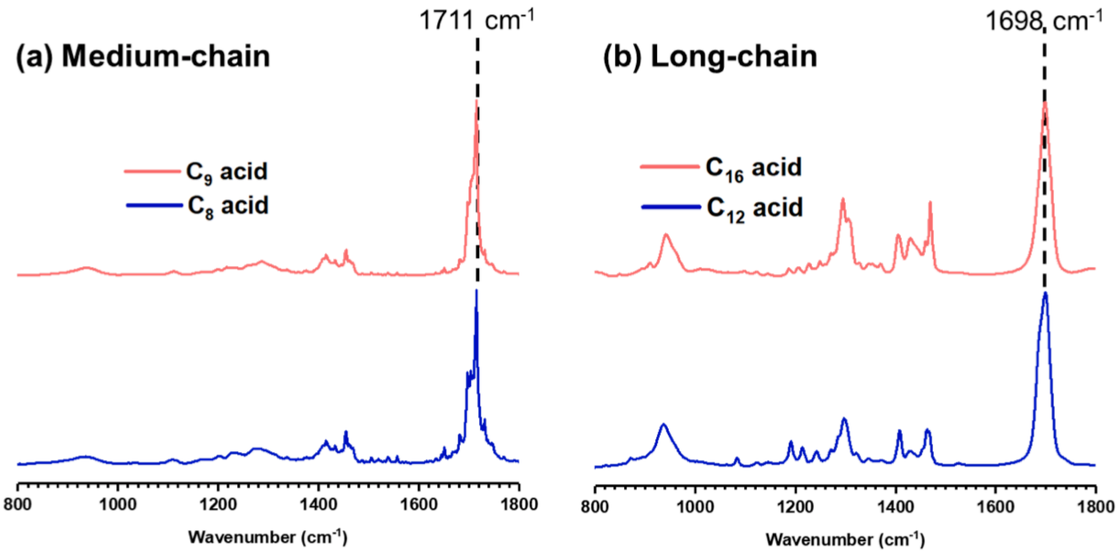

Figure S3. O-PTIR spectra of (a) medium-chain carboxylic acids and (b) long-chain carboxylic acids in methanol dried onto the CaF_2 crystal. Medium-chain exhibits a C=O stretching vibration at 1711 cm^{-1}, indicative of the carboxylic acid functional group while long-chain exhibiting C=O stretching vibration at 1698 cm^{-1} from hydrogen bonded carboxylic acid dimer.

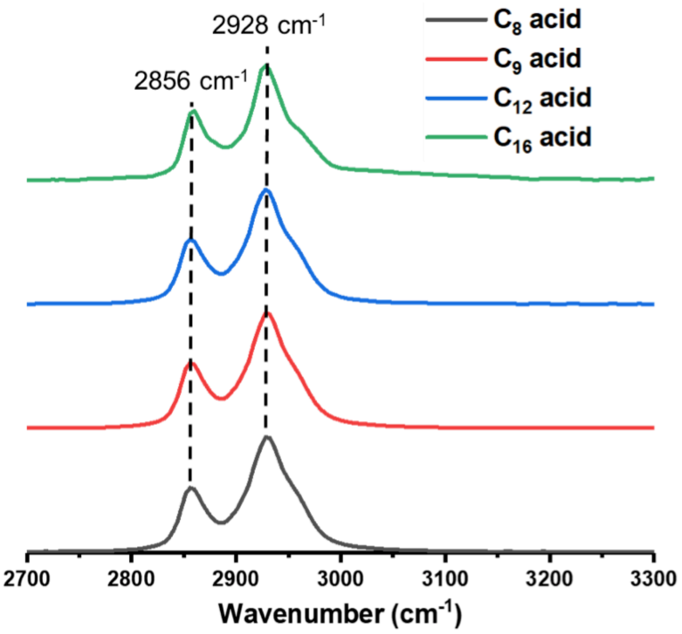

Figure S4. O-PTIR spectra of microdroplets formed from C_8, C_9, C_{12} and C_{16} carboxylic acids in methanol shown in the C–H stretching region with peaks at 2856 and 2928 cm^{-1}. These data were collected using an OPO laser.

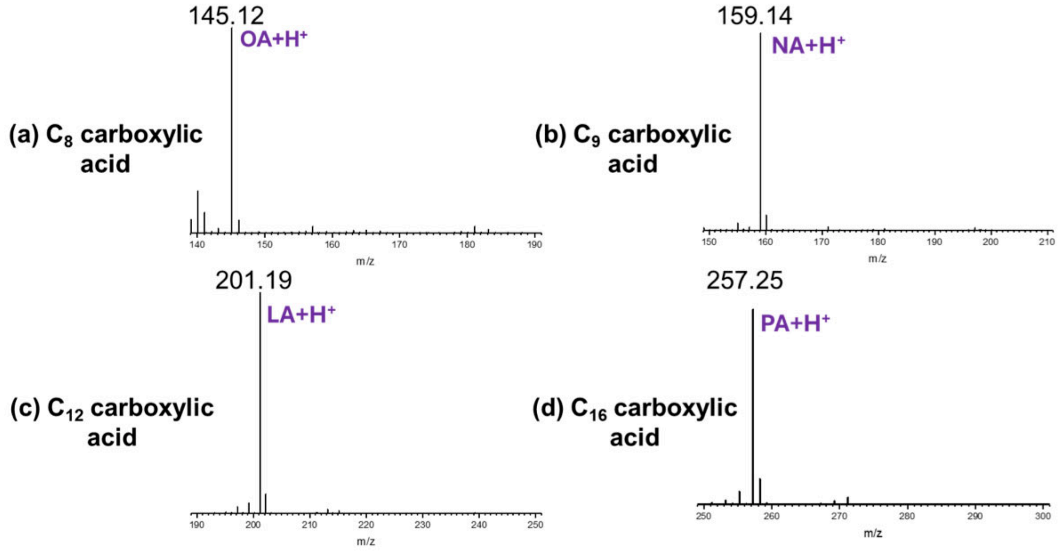

Figure S5. Orbitrap mass spectra of carboxylic acids in acetonitrile: (a) C_8 carboxylic acid with a peak at m/z=145.12, corresponding to octanoic acid (OA + H^+); (b) C_9 carboxylic acid with a peak at m/z=159.14, corresponding to nonanoic acid (NA + H^+); (c) C_{12} carboxylic acid with a peak at m/z=201.19, corresponding to lauric acid (LA + H^+); (d) C_{16} carboxylic acid with a peak at m/z=257.25, corresponding to palmitic acid (PA + H^+).

Supporting Information Table

Table 1. m/z values, chemical formula, and mass errors (in ppm) for different esters formed from reaction of saturated fatty acids and alcohols in microdroplets.

m/z	Chemical formula	Mass error	Ester Product
159.14	$C_9H_{19}O_2$	3.353	C_9 ester from C_8 acid in methanol
173.15	$C_{10}H_{21}O_2$	2.908	C_{10} ester from C_9 acid in methanol
215.20	$C_{13}H_{27}O_2$	2.711	C_{13} ester from C_{12} acid in methanol
271.26	$C_{17}H_{35}O_2$	2.002	C_{17} ester from C_{16} acid in methanol
173.15	$C_{10}H_{21}O_2$	2.677	C_{10} ester from C_8 acid in ethanol
187.17	$C_{11}H_{23}O_2$	2.423	C_{11} ester from C_9 acid in ethanol
229.22	$C_{14}H_{29}O_2$	2.763	C_{14} ester from C_{12} acid in ethanol
285.28	$C_{18}H_{37}O_2$	1.834	C_{18} ester from C_{16} acid in ethanol

REFERENCES

1. Sit, I.; Quirk, E.; Hettiarachchi, E.; Grassian, V. H. 'Differential Surface Interactions and Surface Templating of Nucleotides (dGMP, dCMP, dAMP, and dTMP) on Oxide Particle Surfaces" *Langmuir* **2022**, *38*, 15038–15049.

2. Molina, C.; Kim, D.; Mehndiratta, L.; Lee, J.; Madawala, C.K.; Slade, J.H.; Tivanski, A. V.; Grassian, V. H. "A Comparison of Different Vibrational Spectroscopic Probes (ATR-FTIR, O-PTIR, Raman and AFM-IR) of Lipids and Other Compounds Found in Environmental Samples: Case Study – Substrate-Deposited Sea Spray Aerosols" *ACS Measurement Science Au* **2025**, *5*, 74-86.
